# Tailored physical activity on prescription with follow-ups improved motivation and physical activity levels. A qualitative study of a 5-year Swedish primary care intervention

**DOI:** 10.1080/02813432.2020.1842965

**Published:** 2020-11-11

**Authors:** Monica Joelsson, Stefan Lundqvist, Maria E. H. Larsson

**Affiliations:** a Närhälsan Gibraltar Rehabilitation, Region Västra Götaland, Gothenburg, Sweden; b Department of Health and Rehabilitation, Unit of Physiotherapy, Institute of neuroscience and Physiology, Sahlgrenska Academy, University of Gothenburg, Gothenburg, Sweden; c Centrum för fysisk aktivitet Göteborg, Region Västra Götaland, Gothenburg, Sweden; d Research and Development Primary Health Care, Region Västra Götaland, Gothenburg, Sweden

**Keywords:** Physical activity, metabolic risk factor primary health care motivation, qualitative study

## Abstract

**Objective:**

To explore how physically inactive patients, with metabolic risk factors, experienced long term treatment with physical activity on prescription.

**Design:**

Qualitative content analysis of individual interviews after strategical sampling of respondents.

**Setting:**

Fifteen primary health care centres in Gothenburg, Sweden.

**Subjects:**

Twenty physically inactive patients, with one or more metabolic syndrome components, 9 women, 11 men, mean age 58 years (25–73); 10 patients were responders and 10 non-responders to the intervention.

**Main outcome measures:**

Categories describing treatment effect and successful intervention

**Results:**

The interviews revealed three categories of effect. First, individual adjustments contributed to increased physical activity. Second, follow-up and support were valuable aids for prioritising and maintaining lifestyle changes. Third, motivation could be higher if patients make their own choices and experienced positive health effects. The overarching emerging theme was ‘tailored physical activity on prescription with regular follow-ups can contribute to increased and maintained motivation and physical activity levels.’

**Conclusion** Physical activity on prescription in a Swedish primary care setting was successful when the recommended physical activity and follow up was individually adapted.

**KEY POINTS**
- Individually adapted physical activity on prescription gave insight to increase physical activity levels in a 5-year Swedish primary care intervention directed towards inactive patients with the metabolic syndrome
- Motivation increased for patients designing their own routines for physical activity.
- Experiences of positive health effects helped maintain or increase physical activity levels, and follow-up and support from healthcare professionals helped to prioritise life style changes.

## Introduction

Globally, one-third of all adults are physically inactive [1]. Existing public health guidelines for physical activity recommend a minimum level of 150 min of moderate-intensity or 75 min of high-intensity physical activity per week [2,3]. A 2016 study in Sweden showed that 70% of the population aged 50–64 years did not adhere to the recommended minimum level of physical activity [4,5]. The least physically active individuals have the most to gain from increasing physical activity level and adopting a regular routine [6,7] and remaining physically active from young age or increasing the level of physical activity in middle age may also reduce the risk of future heart failure in healthy women [8]. Being inactive is one of the leading risk factors for lifestyle-related diseases and premature death carrying a 20–30% increased risk for cancer, heart disease, stroke and diabetes, with reduced life expectancy of 3–5 years. Physical inactivity is also associated with increased societal costs through medical care and lost productivity [9]. Metabolic syndrome is associated with physical inactivity, and increased risk for type 2 diabetes and cardiovascular disease [10]. The syndrome is not consistently defined, but includes abdominal obesity, insulin resistance, dyslipidaemia and hypertension in various combinations [11]. Physical activity positively affects all components of the metabolic syndrome [12].

Internationally, healthcare systems have tried various methods to encourage patients to increase physical activity [13]. In several countries, a model called ‘exercise referral schemes’ has been used in which the patient is offered predetermined activities for a specified period [14]. Systematic reviews of exercise referral scheme studies show varying results, with small to medium positive effects on physical activity level, usually concluding with a recommendation for further research [15–20]. Sweden has developed its own model, physical activity on prescription (PAP), which is partly implemented in Swedish health care [21]. Swedish PAP-treatment includes an individualised consultation with a recommendation for physical activity adapted to the patient’s current state of health, motivation level, self-efficacy, and readiness to change. The consultation is often based on the principles of motivational interviewing that originated from the Transtheoretical Model, but this approach is not an absolute requirement in Swedish PAP-treatment [22]. The outcome of the treatment, however, depends on the level of motivation that can be achieved, which is a relevant area of interest.

The level of motivation is based on theories previously described by Prochaska et al. and Deci and Ryan [20,21], and that underlie extensive ongoing research [22–24]. The recommendation for physical activity includes a specified frequency, duration and intensity for the selected physical activity. The patient is also offered an individually adapted follow-up within the health care system [21,23]. In Sweden, PAP-treatment can be used by all licensed health care professionals (e.g. physicians, nurses, physiotherapists, occupational therapists, psychologists) with knowledge about the patient’s health status, the use of physical activity in disease prevention and treatment, and the concept of the Swedish PAP model [24].

A systematic review that evaluated the Swedish PAP model concluded that ’Swedish PAP probably improves the level of physical activity and results in little or no difference in adverse events compared with no PAP‘. These authors recommended that Swedish PAP should be a part of regular healthcare to increase physical activity in patients [25]. Other studies have shown that the Swedish PAP model had positive effects on, or prevented deterioration of cardio-metabolic risk factors and increased health-related quality of life and physical function [26–28]. Adherence to PAP appeared to be as good as or better than adherence to other long-term treatments, including drugs [29]. Compared to oral advice, a written prescription has also proved to be more effective for increasing physical activity level [5,30]. Despite these demonstrated effects, the method remains underused as a treatment strategy in the Swedish health care system, and higher uptake would be expected to benefit public health [29].

People with obesity, heart disease, or chronic pain often have had positive experiences when they increased physical activity; however, consistency in outcomes among relevant studies has been hampered by environmental and social factors, the body experience of the participants, and varying health status [21,31,32]. Previous experiences of physical activity also are an important facilitating factor [24,31]. Moreover, patients who perceived prediabetes (impaired glucose tolerance between normal glucose tolerance and overt diabetes, increasing the risk of type 2 diabetes) [33] as a threatening risk factor showed greater behavioral changes with physical activity [34]. Increasing physical activity might also have positive effects on other behaviors, such as dietary habits [34,35]. Although leisure-time physical activity in adults has increased in recent years in several Nordic countries, the difference in levels of physical activity among socio-economic groups seems to persist, with adults in socioeconomically vulnerable groups being less likely to be physically active [36]. Knowledge is lacking, however, about the different needs of individuals to provide sufficient support in increasing physical activity [31,35,37].

The present study aimed to capture and describe how physically inactive patients with metabolic risk factors experienced PAP-treatment, provided by a health care centre.

## Materials and methods

### Setting and participants

The interview study included 20 of 444 patients who had participated in an ongoing PAP treatment study at 15 primary health care centres in Gothenburg [28,38]. The Gothenburg PAP-study was ongoing when the patients were included in this interview study, and 71 of the 444 patients had completed the 5-year PAP study. The patients were 27–85 years old, had at least one component of the metabolic syndrome, and were insufficiently physically active at baseline, according to the public health recommendation [5,39]. The metabolic syndrome components in this study were classified according to the National Cholesterol Educational Program (NCEP) with cut-off values as follows: waist circumference, *>*88 cm for women and *>*102 cm for men; blood pressure, ≥130/85 mm Hg; fasting plasma glucose, ≥6.1 mmol/l; triglycerides, ≥1.7 mmol/l; and high-density lipoprotein, *<*1.3 mmol/l for women and *<*1.0 mmol/l for men [40]. Of the 20 included patients, 80% had abdominal obesity and hypertension, 60% had hyperlipidaemia, and 30% hyperglycaemia. Fifty-five percent of the patients had more than three metabolic risk factors according to NCEP classification.

#### Intervention

The PAP-intervention has been described in detail previously [28]. During the 5-year intervention, authorised personnel, mainly nurses, at the health care centres offered PAP-treatment containing three core elements: patient-centred consultation about physical activity, individual recommendation of physical activity with a written prescription, and an individually adapted follow-up. Most patients had follow-ups 1–4 times per year either by revisits or telephone contacts containing the three core elements of PAP. The consultation with the patient was often based on the principles of motivational interviewing. Self-assessed physical activity and metabolic risk factors, according to NCEP classification [40,41], were measured at the health care centre once a year.

#### Recruitment

As a first step in the recruitment strategy, five health care centres with permanently employed clinicians who had knowledge and experience of PAP treatment were selected. The second author made the selections, based on knowledge about the health care centres. Participants who had completed the 5-year follow-up were then identified as responders or non-responders, according to whether they had achieved the public health recommendation. Each patient responded to two physical activity questions, in which 30 min of moderate-intensity physical activity per day was worth 1 point and 20 min of high-intensity physical activity per day was worth 1.7 points during each day of the week. A value of <5 points indicated an inadequate physical activity level [39]. Patients with ≥5 points were defined as responders and those with <5 points as non-responders. Of 160 patients included in the main study from the selected health care centres, at the time of inclusion in this interview study, 71 had completed the 5-year follow-up. Of these, 39 were responders, and 32 were non-responders.

In a second step of the recruitment strategy, all participants in the two groups were divided into sub-groups, based on sex, age, and BMI (above or below 30) to obtain the greatest variety possible. Twenty participants in each group received an informational letter about the study. A study administrator contacted the participants 1–2 weeks after the letter was sent to determine their interest in participating in the interview study. Contact attempts were terminated when 10 informants in each group agreed to participate in the study. During the telephone contact time, 10 patients declined to participate in the interview. The participants included nine women and eleven men, with a mean age of 58 years (range 25–73). Of these, nine were responders and eleven were non-responders (participant characteristics are presented in Table 1).

**Table 1.** Characteristics of participants in a 5-year study of physical activity on prescription (*n* = 20).

Sex	Age	BMI
*PA* < 150 min/week		
M	34	33
M	39	33
M	43	32
M	47	46
M	53	33
M	72	29
F	42	31
F	67	30
F	67	35
F	70	29
F	70	31
*PA* > 150 min/week		
M	54	32
M	64	32
M	66	40
M	72	31
M	73	28
F	40	36
F	56	29
F	63	25
F	67	35

#### Data collection

All interviews were conducted in Swedish. All participants received a consent form, included with the information letter that they signed and submitted before the interview.

The interviews were semi-structured with the following open questions:

- What does physical activity mean to you?
- What is your experience with PAP?
- What did it mean to you to receive PAP?
- What has complicated or facilitated your ability to increase your level of physical activity?
- What have you experienced as good, less good, or bad in your physical activity during the PAP-treatment period?

The interviews took place at the PAP-centre, in April and May 2018, administered by the first or second author.

The participants were invited to speak freely during the interview. To clarify each individual’s story, further questions were asked. The interviews lasted 15–-30 min, and were recorded and transcribed verbatim. Formal ethical approval was received from the Regional ethical review board in Gothenburg, Sweden, prior to the study, (Dnr 1139-17).

#### Data analysis

The semi-structured interviews were analysed using a qualitative content analysis approach, with an inductive focus as described by Graneheim and Lundman [42,43]. The purpose of the analysis was to focus on the manifest and latent content of the participant’s thoughts and experiences of PAP-treatment. According to the definition of qualitative content analysis, the visible and obvious components in the unit of analysis, referred to as the ‘meaning units’, form the manifest content. When it comes to abstracting these into categories and a theme, a varying depth of abstraction and interpretation was created that illustrated the latent content.

The unit of analysis was the transcribed text. Each interview was read several times, and meaning units relevant to the purpose were identified. These meaning units then were condensed and coded. All three authors encoded the first three interviews independently after which they discussed each interview to reach an agreement and a common understanding of the coding strategy. The first author encoded the remaining interviews, after which the categorisation procedure was begun.

For this process the codes were sorted into subcategories, based on similarities and differences. Content conformity in the subcategories was verified and revised in discussion with the second author.

In the next step, the subcategories were sorted and abstracted into categories that were discussed and revised in an iterative, comprehensive, and reflective process among all authors until consensus was reached. Finally, there was an agreement and formulation of an overall latent theme, according to the underlying significance. Examples of the meaning units, condensed meaning units, codes, subcategories, and categories are provided in Table 2.

**Table 2.** Examples of analysis process with meaning unit, condensed meaning unit, code, subcategory and category.

Meaning unit	Condensed meaning unit	Code	Subcategory	Category
You get a puff, or a kick that I have to do this. If you look at the PAP you feel that it is a need. I can see my test results	The test results on the paper gives you a kick to start	PAP gives you a kick	PAP becomes an eye opener	Individual adjustment contribute to increased physical activity
Each time when you were going on follow-up you were spurred, first before, you sharpened up and when leaving you were extra motivated	You sharpened up before and were more motivated after the follow-up	Follow-up makes you more motivated before and after	Follow-up reminds and motivates	Follow-up and support are valuable aids in for prioritising and maintaining Lifestyle changes
I think that if you’re not regular in your activities, you do not get the effects that you want	If you’re not regular in your activities you don’t reach the effects	Regularity is necessary to have effect	Routines facilitate	Motivation can be higher if patients make their own choices and experience positive health effects

The software program NVivo 11 Software 2015 (QSR International Pty Ltd) was used to organise data in the analysis process.

## Results

The analysis identified three categories, as follows: (1) Individual adjustments contribute to increased physical activity; (2) Follow-up and support are valuable aids for prioritising and maintaining lifestyle changes; and (3) Motivation can be higher if patients make their own choices and experience positive health effects. The emerging, latent, and overarching theme that illuminated participant experiences with PAP was articulated as follows: *Tailored physical activity on prescription and regular follow-up contribute to increasing and maintaining motivation and level of physical activity.*

**Figure 1.**
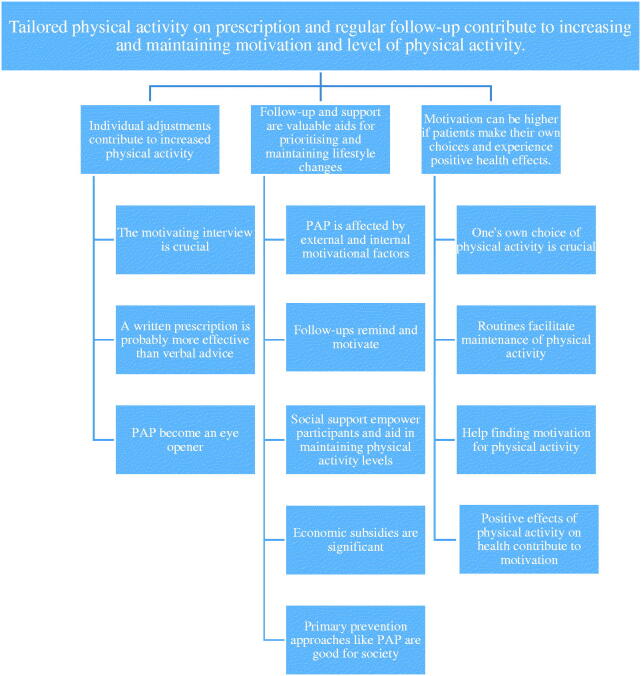
The image shows the results from the analysis process and is read from below. Participants’ experiences of PAP-treatment were coded and sorted into different subcategories. The subcategories were then grouped into three different categories and finally an overall theme was created.

The theme, categories and subcategories are presented in Figure 1.

#### Individual adjustment contributes to increased physical activity

This category described the importance of conforming the PAP-treatment to the participant’s present situation and previous experiences of physical activity and ensuring that the chosen physical activity was perceived as sufficiently attractive. The health care provider could then provide examples of where the patient could find such an activity. Finally, an agreement was reached on a start date for the selected physical activity.

##### The motivating interview is crucial

It was important that the dialogue with the health care provider was perceived as respectful throughout the treatment period. The recommended physical activity had to be individualised regarding the level of motivation, previous experiences of physical activity, and possible limitations. The participants described the need to be confirmed, to receive a detailed description of the PAP concept, and to be informed about possible health effects. Furthermore, they viewed it as crucial to have a clear plan for when, where, and how the physical activity should start and continue. The participants experienced this procedure as an agreement or a duty that they wanted to uphold. As one participant put it:

It is not always just giving out a prescription and then expecting the person to start, because one has to see the person, how the person is feeling and so on. (ID14)

##### A written prescription is probably more effective than verbal advice

The participants expressed different perspectives on the written prescription. Some saw it as a reminder, some took it more seriously, and some became more motivated. It was also useful to have a written prescription to show when entering the gym. A written prescription was less important for participants who were already highly motivated, had previous experience of physical activity, or believed that physical activity was their own responsibility. Of having the written prescription, one participant said:

I probably took it more seriously. (ID5).

##### PAP becomes an eye opener

The participants stated that they had expected pharmacological treatment, not PAP-treatment, when they made an appointment at the health care centre.

Thus being offered PAP-treatment often came as a surprise to them, although they had heard about the potentially positive health effects of increased physical activity and even had previous experiences of these effects. In combination with a newly discovered metabolic risk factor, this insight contributed to increased motivation, and PAP became a motivator to further increase physical activity level. PAP also resulted in new social contacts that provided support for increased physical activity. Some participants were not motivated by PAP, and the discovery of a metabolic risk factor made them feel disappointed or depressed. However, continued follow-ups from the health care provider contributed to a sense of support and security and motivated them to continue in the study.

…good to see how the cholesterol change, i.e. how it is raised and lowered and what effects those changes have. (ID18).

It was difficult to receive the negative results of the screening. (ID11)

#### Follow-up and support are valuable aids for prioritising and maintaining lifestyle changes

The participants emphasised a need for regular follow-ups to maintain motivation because the follow-ups provided a visualisation of the effects of metabolic risk factors on health.

The importance of different kinds of support, e.g. from family, friends, or professionals was also emphasised.

##### PAP is affected by external and internal motivational factors

Both internal factors (e.g. a sense of duty, shame, or insurmountable effort) and external factors (e.g. weather conditions, lack of money or time) were described as affecting adaptations to the recommended physical activity. The participants were either aware that they prioritised other things or that other factors obstructed their ability to perform physical activities. Common examples of obstacles to implementing physical activity were an aversion to physically hard work, bad weather conditions, lack of time, musculoskeletal pain, or limiting disease.

It may be that it one day rains like hell on a given day, and then I do not want to take that half hour walk to the gym. (ID12)

I had to quit since my daughter was a single mother and very much needed help, and I simply felt that it became too much around me. (ID8)

##### Follow-ups remind and motivate

The participants appreciated and desired more follow-ups for an extended period of time. The follow-ups served as an extra motivating factor and an opportunity for ongoing support in upgrading or continuing the level of physical activity. The follow-ups also included a control of the metabolic values that triggered the prescription, which contributed to a high degree of perceived safety. Contact with the healthcare provider was described as very positive and was a strong contributing factor in achieving sustainable change in physical activity. Participants also expressed that support from a physiotherapist would be more appropriate than support from a nurse, when increasing physical activity and receive individualised dosage.

Each time you went for a follow-up, you left more motivated. (ID3)

##### Social support empower participants and aid in maintaining physical activity levels

The participants stated that a change in physical activity posed new demands on them, their friends, and their relatives. People who surrounded the participant helped remind them and provided additional encouragement to maintain the physical activity. Moreover, through physical activity, new friends could be found who contributed to new habits. Group exercise was sometimes appreciated, either conducted by a trainer or practised with a friend or partner. The environment was also perceived as important, e.g. living near a training site or a nature area.

I met like-minded individuals and peers, and we had a lot of fun and therés a good leader there. This worked really well, and the guys also said: "Do you want to join us at the gym?" “Yes, I can do that,” I answered. And then, when I got there, we did circuit training, both women and men. (ID14)

##### Economic subsidies are significant

The participants appreciated that the written prescription sometimes contributed to reducing the cost of joining an exercise facility; otherwise, the fee might have been an obstacle. However, some commented that the cost should be adapted to the participant’s finances, that a certain amount of money should be distributed among non-discounted activities, or that they should be given the opportunity of a trial period before buying an expensive gym membership. On the other hand, it was mentioned that an expensive membership can make the individual feel more obliged to use it.

It is wonderful that these prescriptions are available, and that you can buy discounted gym memberships. (ID14)

Going to the gym can be very expensive. (ID14)

##### Primary prevention approaches like PAP are good for society

PAP has raised awareness of the effects of and need for physical activity. PAP was considered important for preventative purposes and for reducing the increasing costs of health care.

To make sure that people actually exercise, it still has to be better for public health and reduce healthcare costs in the future. (ID13)

#### Motivation can be higher if patients make their own choices and experience positive health effects

The participants felt that their own opinions, wishes, and integrity were important factors in the process of finding a suitable physical activity. The health care provider supported participants in creating new routines and in increasing their knowledge about the positive effects on physical and mental health. This support made it easier to maintain motivation.

##### One’s own choice of physical activity is crucial

The participants expressed the importance of finding a physical activity that felt appealing, motivating and functional in their everyday life schedule. Therefore, to help the participant make a suitable, realistic choice, the health care provider needs knowledge about various physical activity options. Although motivation level increased, participants could feel trapped by having to adapt to a specific training schedule. In those cases, being able to plan and create one’s own physical activity routines contributed to a feeling of freedom.

I feel a sense of freedom because I do not have to go in on Monday morning, if I'm going to do something else, but at the same time, I do try to maintain a routine. (ID2)

##### Routines facilitate maintenance of physical activity

Routines and regularity were important factors that contributed to continuing the exercises. Routines also made it easier to prioritise physical activity and to avoid getting distracted by non-physical activities. Participants stated that exercising immediately after work, or having scheduled exercise occasions facilitated physical activity routines.

Once you have decided that now, we will do it every Tuesday, then it will be every Tuesday. I think that's good because I have a very active life. (ID19).

##### Help finding motivation for physical activity

Participants who were not accustomed to physical activity or were insufficiently motivated expressed the need for help in finding motivation. In addition to the first motivational consultation with the health care provider, they desired a recurring telephone call or a supervisor who noticed when they had not attended training sessions.

They don’t need to do anything extra, other than just literally kick my ass, maybe chase me more, or simply make a phone call and ask - “How are you doing?”… Then maybe I could get started more easily. Maybe freedom comes with responsibility? But freedom may also reduce responsibility … (ID15)

##### Positive effects of physical activity on health contribute to motivation

The presence of metabolic risk factors was described as another motivational factor and a sufficiently important reason to cross the threshold to a more physically active lifestyle. The positive effects of increased physical activity on health contributed to an increasing motivation to exercise.

I feel healthier, both physically and mentally, because it is also important for mental well-being; I feel good about myself when I have followed through … When I do not, I'm upset with myself for cheating. (ID13)

## Discussion

### Principal findings

The main finding was that most of the participants in the present study expressed satisfaction with the tailored prescription and regular follow-up by skilled health professionals. A lifestyle change requires a certain degree of motivation. The purpose of Swedish PAP-treatment is to increase motivation through support in the form of consultations and follow-ups. In the present study, motivation appeared to increase when the prescription was defined in collaboration between the participant and the health care professional during the consultation. The individualised consultation might have contributed to increasing the participant’s awareness of the positive effects of physical activity on health, which then enabled a change in lifestyle. These factors might have led to increases in the intrinsic motivation, i.e. a sense of autonomy, competence, and control. This interpretation is consistent with self-determination theory, which explains motivation as multidimensional and the result of a combination of diverse factors. The theory postulates that behaviour may be intrinsically motivated, extrinsically motivated, or amotivated, meaning that there is a variation in level of self-determination, i.e. a sense of freedom to make your own choices. The types can be described as fluctuating on a continuum of self-determination in which the intrinsic motivation represents the highest levels of autonomy, the extrinsic motivation is at a mid-level and the amotivation is at the lowest level [44]. Non-individualised counselling might constitute a risk for undermining the participant’s inherent motivation if it is perceived as an attempt to control behaviour [44]. This factor is illustrated in the category; *‘Individual adjustments contribute to increased physical activity’.*

Another contribution to motivation might have been to make the prescription visible in the home to serve as a reminder. As mentioned previously, compared to oral advice, a written prescription has shown to be more effective for increasing physical activity levels .

The participants expressed the need for different types of support, such as recurring follow-ups and regular contact with the healthcare provider, over a long period of time to maintain motivation. Support from family, friends, and other physical activity participants also increased motivation and contributed to maintaining physical activity routines. This finding is consistent with the results of a study by Lidegaard et al. [45], who found that participants felt that physical activity with others contributed to a high degree of motivation. The participants also requested support in the form of group training, preferably with supervision. Previous studies have found that participants with chronic diseases prefer to exercise in training groups under safe conditions and with supervision from a healthcare professional [31]. Even when the participants were aware of their own responsibility regarding health, they wanted support, professional assessments, and accessibility to health care [31]. In the present study, participants also stated that they would feel more support from a physiotherapist when increasing the physical activity level. Another form of support was financial subsidy. Training in a gym was often considered too expensive, and cost could become a limiting factor. Romé et al. have also found that an individual’s economic assets can affect level of motivation and prevent the implementation of lifestyle changes [46].

Consequently, all forms of subsidies were considered positive, and in the study area, a large city, there were more opportunities for subsidies or free-of-charge physical activity compared to smaller municipalities and rural areas [47,48]. Comments about follow-ups and different kinds of support were gathered into the category, ‘*Follow-up and support are valuable aids for prioritising and maintaining lifestyle changes’*.

Some participants stated that PAP-treatment did not result in any change in physical activity, although they were aware of the health-promoting effects of increasing physical activity. According to Proschaska and DiClemente’s transtheoretical model, changes occur in steps that are not always visible to other people [22]. The first stage is denial; one does not believe that changing behavior will have any effect, or one simply has no desire for change. The second step is contemplation, in which one begins to consider that, as in the present study, physical activity might favourably influence the metabolic risk factor. Potentially, with this step, the patient moves closer to a change in lifestyle, although without any change in activity level. In the following steps of the model, the changes become more obvious to other people.

In recent years, there has been a general change in social attitudes about the importance of physical activity. Many people are aware of the positive effects of physical activity, even though some do not have the motivation, the will, or the opportunity to make a change.

The likelihood of behavioural change increases when the participant takes an active role in the decision making and greater responsibility for the process [44]. This value in ownership was described in the category; *’Motivation can be higher if patients make their own choices and experience positive health effects’.* When a change in behaviour was incorporated into a routine and the participants experienced positive physical and psychological effects, motivation was enhanced and the possibility of maintaining a permanent change increased. Lidegaard et al. also described this effect. They showed that visible health indicators, such as improved blood sugar levels, became a motivating factor because they generated a sense of immediate reward [45]. However, these results conflicted with those from a study of patients with obesity, in whom monitoring was sometimes detrimental to patient motivation [49].

In a previous study, chronic pain emerged as a possible recurrent barrier to physical activity, because of difficulties in creating a physical activity routine when the pain varied and took control of everyday life [31]. In the present study, the participants were looking forward to the follow-up at the health care centre to find out whether their metabolic risk factor values had changed. The discovery of a metabolic risk factor appeared to increase the level of motivation, i.e. the sign of disease might have become a facilitator for increasing physical activity. Moreover, some participants began to challenge themselves to improve on previous abilities. This outcome is in line with the results from a study by Miettola and Viljanen, who found that individuals with poor health awareness had a greater need for professional support in lifestyle changes, and those with practical obstacles in their daily lives were aided more by social support. Individuals with a strong sense of coherence i.e. with the ability to adapt to a complex context only needed encouragement to maintain a healthy lifestyle [50]. It is also interesting that the patients seemed to appreciate the offered the PAP intervention, regardless of their actual change in physical activity.

### Strengths and weaknesses of the study

The concepts of credibility, confirmability, dependability, and transferability of the findings are discussed below in regard to the study’s trustworthiness [51]. These tenets have also previously been described by Graneheim and Lundman [42,43].

Choosing participants with various experiences of physical activity increased the opportunity to see the research question from a variety of aspects. The interview was based on five open questions, which the interviewer clarified if necessary by asking supplementary questions. Then the interviews were performed and transcribed in Swedish. English translations were made when the meaning units were determined. This translation might have affected the information.

Regarding confirmability, there was a risk of social desirability bias. The interviewers were not known to the informants before the study, but on request, they presented themselves as researchers and physiotherapists working in the healthcare sector. The interviewers were, however, not involved in either the PAP-treatment or the recruitment process, which reduced the risk of bias. Moreover, the research questions were grounded in clinical empirical work, which could have affected the interview questions, data collection, and data interpretations. To mitigate this potential limitation, the first three interviews were coded independently by all three authors, and differences were discussed to reach an agreement and a common understanding of the coding strategy. Finally, all of the categories were discussed and revised in an iterative process among all the authors. To strengthen the credibility and dependability, the co-authors continuously strived to reach a consensus about the analysis and interpretation. Direct quotes from the interviews were also presented in the manuscript to illustrate the results and ensure these tenets.

To facilitate transferability, we used Table 1 and the text in the Methods and materials section to describe details of the selection progress, patient characteristics, the data collection process, and the analytical procedure.

Of note, the cohort included more men than women, and the men were slightly younger than the women.

All participants included in this study resided in Gothenburg, the second largest city in Sweden. Thus, they had the advantage of living near training facilities and recreation areas, which might have affected their motivation and ability to maintain the physical activity [52].

### Findings in relation to other studies

This study highlighted the need for an individually customised PAP-treatment with professional support from skilled health care providers for physically inactive patients with metabolic risk factors. The participants stated that learning that they had a metabolic risk factor increased their motivation for changing their physical activity levels, which differs from patients with chronic pain or obesity [31,49]. A variation of PAP methods or exercise referral schemes have been studied in the health care system in various countries [13,16] often with fixed short-term interventions (10–12 weeks) and linked to predetermined activities [18,19,53]. Compared to Swedish PAP method, these interventions may have been less individualized for the patient’s needs and requests, resulting in varying results regarding e.g. physical activity level [54].

### Meaning of the study

The results of the present study indicated that PAP-treatment should be individualised and that recurrent support is appreciated and contributes to an increased level of physical activity. Participants expressed satisfaction with the individually tailored PAP-treatment provided by competent healthcare professionals. They also gained insight regarding the positive health effects of increased physical activity.
